# An improved gene expression programming algorithm for function mining of map-reduce job execution in catenary monitoring systems

**DOI:** 10.1371/journal.pone.0290499

**Published:** 2023-11-16

**Authors:** Jin Ding, Tianyu Jiang, Ping Tan, Yi Wang, Zhenshun Fei, Chuyuan Huang, Jien Ma, Youtong Fang

**Affiliations:** 1 School of Automation and Electrical Engineering, Zhejiang University of Science and Technology, Hangzhou, China; 2 College of Electrical Engineeringg, Zhejiang University, Hangzhou, China; UNITEN: Universiti Tenaga Nasional, MALAYSIA

## Abstract

Gene expression programming (GEP) is one of the most prominent algorithms in function mining. In order to obtain a more accurate function model in configuration parameters-execution efficiency (CP-EE) of map-reduce job in the high-speed railway catenary monitoring system, this paper proposes a novel algorithm, called GEP based on multi-strategy (MS-GEP). Compared to traditional GEP, the proposed algorithm can escape premature convergence and jump out of local optimum. First, an adaptive mutation rate is designed according to the evolutionary generations, population diversity, and individual fitness values. A manual intervention strategy is then proposed to determine whether the algorithm enters the dilemma of local optimum based on the generations of population evolutionary stagnation. Finally, the average quality of the population is changed by randomly replacing individuals, and the ancestral population is traced to change the evolutionary direction. The experimental results on the benchmarks of function mining show that the proposed MS-GEP has better solution quality and higher population diversity than other GEP algorithms. Furthermore, the proposed MS-GEP has higher accuracy on the function model of CP-EE of high-speed railway catenary monitoring system than other commonly used algorithms in the field of function mining.

## 1 Introduction

The real-time nature of map-reduce job execution in high-speed railway catenary monitoring software systems [1] is a key factor in measuring its performance. The execution efficiency of map-reduce jobs greatly impacts the effectiveness of extracting valuable information. The measure of execution efficiency is execution time, for the same job, shorter execution time results in higher execution efficiency. Among the factors that affect the execution efficiency of the map-reduce job, the configuration parameters of the map-reduce job have played a significant part [2]. Due to increasingly complex operational environments and high demands, the traditionally manual optimization of configuration parameters and tuning them based on experience can become difficult [3]. In the context of artificial intelligence (AI), it is feasible and smarter to find the appropriate better parameters by establishing a model of the configuration parameters-execution efficiency (CP-EE) of the map-reduce job. Therefore, it is essential to establish a more accurate and reliable map-reduce job CP-EE function model.

Recently, several attempts have been made to extensively study the optimization of the big data job configuration parameters. Consequently, several authors proposed many models for the execution efficiency of the big data job using AI methods. Vidhyasagar et al. [4] searched for the optimal cluster configuration using the opposing chaotic flower pollination algorithm within the parameter auto-tuning system. Xiaoling Luo et al. [5] proposed a performance optimization method for Hadoop cluster systems using a simulated annealing algorithm. Ali Khaleel et al. [6] obtained the optimized Hadoop cluster configuration using a genetic algorithm (GA) [7] and a novel intelligent algorithm based on genetic programming (GP) [8], which achieved optimal performance on MapReduce programs. Bei et al. [9] first developed a performance prediction model for each Hadoop component using random forest (RF) regression algorithm, and searched for the optimal parameters using GA.

Gene expression programming (GEP) [10] is an evolutionary algorithm borrowed from GA and GP. Traditional AI regression models [11] require predefined function structures such as linear and polynomial regression in machine learning, and also tend to be more difficult to explain their internal decision-making processes such as neural networks. GEP generates expressions that are mathematical forms or procedures that make it easier to explain and understand how the model works, helping to reveal patterns and relationships in the data. It has been widely applied in classification [12], clustering [13], time-series data prediction [14], and function mining [15]. The ratio of the highest fitness of the population individuals to the maximum fitness in GEP determines the accuracy of the mined function model. However, the standard GEP still tends to trap the population evolution in a local optimum, preventing the algorithm from obtaining a more accurate function model.

Therefore, this paper proposes a GEP algorithm based on multi-strategy (MS-GEP) for CP-EE of the map-reduce job function mining model construction in the catenary monitoring system of high-speed railway and some shortcomings of GEP algorithm. The proposed MS-GEP can obtain a more accurate function model using the GEP algorithm optimized by the adaptive mutation rate based on population information entropy and manual intervention strategy to guide the population evolution. The main contributions of this paper are summarized as follows:

(1) We propose an adaptive mutation rate setting based on the population information entropy that can maintain the diversity of the population by taking it into consideration.
(2) We propose a manual intervention strategy since the traditional GEP fails to converge to a better solution due to converging prematurely or falling into the local optimum.
(3) The experimental results on the benchmark dataset show that the proposed MS-GEP outperforms the other GEP algorithms.
(4) In the function mining experiments of map-reduce job CP-EE of the high-speed railway catenary monitoring system, the evaluation index of the proposed MS-GEP outperforms other commonly used function mining algorithms.

The remainder of this paper is organized as follows. Section 2 introduces the relevant fundamentals. Section 3 presents details of the proposed MS-GEP. Section 4 shows the experimental and analysis results. Finally, Section 5 presents the conclusion and future research.

## 2 GEP

GEP encodes individuals as fixed-length linear strings and then represents them as nonlinear entities of different lengths and shapes. GEP improves the functional complexity loss of individuals caused by fixed-length linear strings during evolution using GA and also optimizes the limited variation of individuals caused by nonlinear entities of different lengths and shapes during evolution using GP. Additionally, GEP has a flexible genetic structure to avoid wasting many resources to check the survival of individuals in GA and GP.

GEP consists of individual coding methods, individual fitness evaluation function, initial populations and genetic operations. The genetic operations consist of selection, mutation, inversion, insertion, gene transformation, and gene recombination. The standard GEP algorithm flow is shown as follows: (1) Creating initial population and parameters setting. (2) Chromosome decoding. (3) Evaluating fitness. (4) Determining whether the end condition is satisfied. If it is satisfied, the process is terminated; otherwise, the next step is executed. (5) Saving the best individuals. (6) Individual selection algorithm. (7) Genetic operation. (8) Forming a new population and return to (2).

Individuals in GEP are called chromosomes. A chromosome can be composed of one or more genes. The combination of gene head and tail forms a gene. The head is made up of a function set and a terminal set, whereas the tail is only made up of a terminal set. Eq (1) calculates the gene tail length *L*_*t*_, where *L*_*h*_ is the gene head length and *n* is the maximum number of operations of a single function in the function set.

$$\begin{array}{c} L_{t}=L_{h}\left(n-1\right)+1 \end{array}$$ (1)

**Fig 1:**
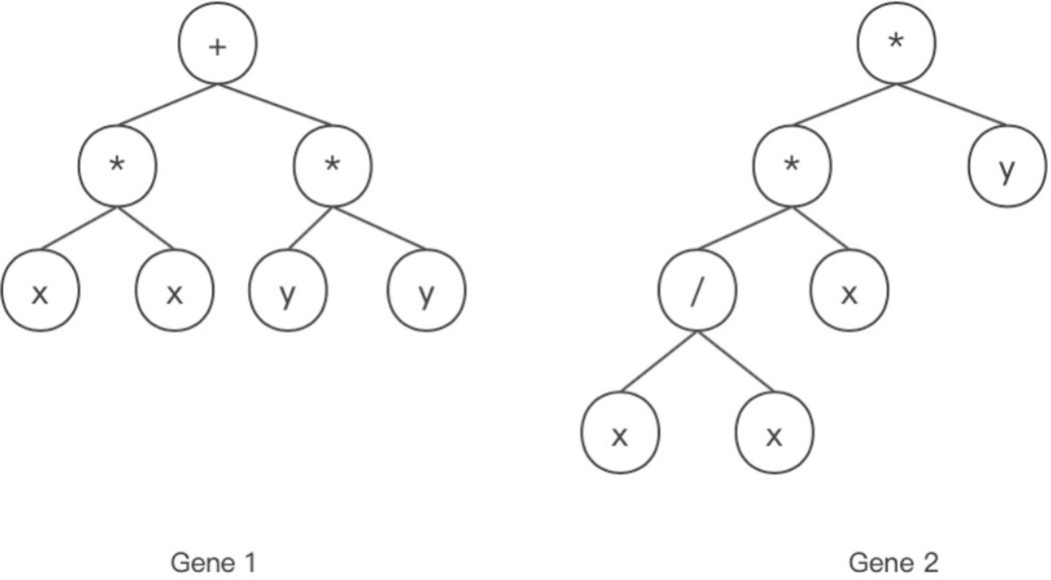
Individual expression tree.

The expressions of chromosomes are divided into expression trees and K-expressions. For example, let the function set be *F* = {+, −, *, /} and the terminal set be *T* = {*x*, *y*}. The length of the gene head is 3. The chromosome consists of two genes. The gene tail length can be obtained as 4 according to Eq (1). The K-expressions of the genes are “+ * *xxyy” and “* *y/*xxx*.” The expression tree is read from top to bottom, left to right to become algebraic. Fig 1 shows the expression tree, gene 1 and gene 2 can be converted to algebraic expressions as “*x*^2^ + *y*^2^” and “*xy*,” respectively. Gene 1 and gene 2 are linked by default with a plus sign. The K-expressions and expression tree can be converted to algebraic expressions as “*x*^2^ + *y*^2^ + *xy*.”

## 3 GEP based on multi-strategy

### 3.1 Adaptive mutation rate setting based on population information entropy(MS-GEP-A)

In GEP, the mutation operation is more effective than the remaining genetic operations in the population evolution [16]. Therefore, the mutation operation plays an essential role in maintaining population diversity, to make the mutation rate setting particularly important. In the standard GEP, the mutation rate is the initial setting parameter. If the mutation rate is too high, the outstanding individuals in the population will be easily destroyed in the late stage of population evolution. If the mutation rate is too low, a large-scale search cannot be conducted in the early stages of population evolution. A high mutation rate is required in the early stages of GEP evolution to facilitate large-scale population search, whereas a low mutation rate is required in the late stages of evolution to avoid the destruction of the population’s best individuals. As demonstrated by previous adaptive mutation rate studies, only individual fitness values or individual fitness values combined with evolutionary generations are usually considered for mutation rate settings without considering the population diversity. To address the above problems, we propose an adaptive mutation rate based on population information entropy by considering the convergence period, population diversity and individual fitness in the mutation rate setting.

Population information entropy is used to show the magnitude of contemporary population diversity, as shown in Eq (2).

$$\begin{array}{c} H=-\sum_{i=1}^{n}P_{i}logP_{i}\;,\;\left(P_{i}\geq 0,\sum_{i=1}^{n}P_{i}=1\right) \end{array}$$ (2)

where *P*_*i*_ is the probability of chromosomes that appear in each region and *n* is the population size. The step for calculating *P*_*i*_ is as follows:

Step 1. The current population individual minimum fitness is *f*_*min*_, the individual maximum fitness is *f*_*max*_, and the population size is *K*. Divide *f*_*min*_ and *f*_*max*_ into *K* regions; each region interval is given in Eq (3).

$$\begin{array}{c} \theta =\frac{\left(1+\beta \right)f_{max}-\left(1-\beta \right)f_{min}}{K} \end{array}$$ (3)

Each interval is then [(1 − *β*)*f*_*min*_ + (*w*1)*θ*, (1 − *β*)*f*_*min*_ + *wθ*), where *w* = 1, 2, ⋯, *K*.

Step 2. Calculate the number of chromosomes appearing in each interval *K*_*i*_. The probability of chromosome that appears in each region *P*_*i*_ is then Eq (4).

$$\begin{array}{c} P_{i}=\frac{K_{i}}{K}\;,\;i=1,2,\cdots ,K \end{array}$$ (4)

Step 3. Using Eq (1), the contemporary population information entropy size *H* is calculated by choosing e as the base.

The adaptive mutation rate can be defined as a nine-tuple: *P*_*auto*_ = {*α*, *p*_*max*_, *p*_*min*_, *H*, *g*, *G*, *f*, *f*_*max*_, *f*_*min*_}, and the redefined mutation rate is shown in Eq (5).

$$\begin{array}{c} \begin{array}{cc} & P_{auto}=P_{min}+\left(P_{max}-P_{min}\right)*exp\left[-\alpha *H*\frac{2f}{f_{max}+f_{min}}*\frac{g}{G}\right] \end{array} \end{array}$$ (5)

where *P*_*max*_ is the upper limit of mutation rate, *P*_*min*_ is the lower limit of mutation rate, *g* is the current number of evolutionary generations, *G* is the total number of evolutionary setting generations, *α* is the correction factor, H is the current population information entropy, *f*_*max*_ is the highest fitness value of population individuals, *f*_*min*_ is the lowest fitness value of population individuals, and *f* is the current individual fitness value.

In the adaptive mutation rate evolution based on population information entropy, the individual mutation rate is less affected by the population information entropy in the early stage of evolution. Individuals in the population can perform large-scale search with large mutation rates and quickly search for better individual’s near the current individual’s location. In the middle and late stages of evolution, the individual mutation rate is more influenced by the population information entropy. When the population information entropy decreases, the population diversity decreases, and the mutation rate increases, increasing the possibility of jumping out of the local optimum in the population evolution. Meanwhile, when the population information entropy increases, the population individuals are more evenly distributed, and the mutation rate decreases, decreasing the possibility of destroying the excellent individuals in the population. Individuals with higher fitness in the population evolve with a relatively lower mutation rate to ensure the succession of high quality individuals as much as possible. In contrast, individuals with lower fitness evolve with a higher mutation rate, which can improve the quality of inferior individuals in the population. Therefore, the adaptive mutation rate setting based on information entropy avoids the premature maturation phenomenon to a certain extent, improves the population diversity, and allows the algorithm to converge to a better solution.

### 3.2 Manual intervention strategies to guide population evolution (MS-GEP-I)

Although the standard GEP can jump out of the premature faster by combining the benefits of GA and GP, the GEP algorithm is irreversible in the evolutionary process, making it unable to easily jump out of the local optimum and search for a better solution when it is stuck in a local optimum dilemma. In GEP, increasing the mutation rate and population size can reduce the possibility of being placed in a local optimum dilemma. Moreover, increasing the mutation rate tends to destroy the good individuals in the population making it difficult for the algorithm to converge stably. The time complexity of the algorithm increases as the population size grows. Thus, the manual intervention strategy is proposed to guide population evolution toward a better solution. Specifically, different intervention strategies are chosen by evolving stagnation generation to jump out of the local optimal as soon as possible. The detailed steps of the manual intervention strategy are shown as follows.

Step 1. In the population evolution process, the first generation of the population after the change in the highest adapted individuals is used as a backtracking point. As the population evolves, the set *BS* is formed from different backtracking points to set up the stack. The alternate set *AS* is set with another initialized population.

Step 2. *islimit*_*a*_ is a judgment that the number of stagnation generations *c*_*a*_ of manual intervention strategy 1 reaches the threshold of population stagnation generations *l*_*a*_, i.e., a judgment that the population evolves into a local optimum in Step 3. *islimit*_*b*_ is the judgment that the stagnation algebra *c*_*b*_ of manual intervention strategy 2 reaches the threshold of population stagnation algebra *l*_*b*_, i.e., the judgment that the population evolution enters the local optimum in Step 4. When *islimit*_*a*_ and *islimit*_*b*_ are false, Step 3 is executed. Meanwhile, when *islimit*_*a*_ is true and *islimit*_*b*_ is false, Step 4 is executed. Furthermore, when *islimit*_*a*_ is false and *islimit*_*b*_ is true, Step 5 is executed.

Step 3. GEP evolves with an adaptive mutation rate based on population information entropy. When the optimal individual fitness of population individuals is not updated, *c*_*a*_ is self-increasing with a step size of 1; otherwise, *c*_*a*_ is reset.

Step 4. Execute manual intervention strategy for contemporary populations 1. Calculate the mean value of individual fitness of the current population, *f*_*avg*_, and calculate the mean value of the individual fitness of current population in ideal condition, *f*_*eavg*_ as shown in Eq (6). Introduce the error factor of fitness value *d*. Calculate *f*_*avg*_ − *f*_*eavg*_.

$$\begin{array}{c} f_{eavg}=\frac{f_{max}+f_{min}}{2} \end{array}$$ (6)

If |*f*_*avg*_ − *f*_*eavg*_| < *d*, the population evolves according to step 3; otherwise, individuals of the contemporary population are randomly replaced according to their parents and grandparents, except for the highest fitness individuals until the condition |*f*_*avg*_ − *f*_*eavg*_| < *d* is satisfied. When the fitness of the optimal individuals of the population is not updated, *c*_*a*_ and *c*_*b*_ are self-increasing in a step size of 1; otherwise, *c*_*a*_ and *c*_*b*_ are reset.

Step 5. Execute manual intervention strategy 2 on the contemporary population and introduce discrimination factor *l*_*p*_ as in Eq (7). Replace all but the highest adapted individuals in the contemporary population according to the discrimination factor. The population *new*_*p*_ is as then shown in Eq (8).

$$\begin{array}{c} l_{p}=\left\lfloor \frac{C_{b}}{l_{b}}\right\rfloor \end{array}$$ (7)

$$\begin{array}{c} New\_p=\{\begin{array}{cc} BS[l_{s}-l_{p}] & l_{p}\leq l_{s}\\ AS[0] & l_{p}\geq l_{s} \end{array} \end{array}$$ (8)

If the discrimination factor does not exceed the length of backtracking point *l*_*s*_, all contemporary populations, except the highest adaptation individuals, are replaced with individuals of the population indexed as *l*_*s*_ − *paralimit* in backtracking point *BS*. Meanwhile, if the discrimination factor exceeds the length of backtracking point *l*_*s*_, all contemporary populations, except the highest adaptation individuals, are replaced with individuals of the population in the alternate set *AS*. When the optimal individual fitness of the population individuals is updated, *c*_*a*_ and *c*_*b*_ are reset.

Introducing the manual intervention strategy has the following benefits. The population evolutionary stagnation generation threshold can be set after introducing the manual intervention strategy 1. When the population cannot jump out of a local optimum in the adaptive mutation rate evolution, individuals of the current population would be randomly replaced by individuals of the parent and grandparent generations to improve the population diversity and the average quality of the population. If the population is still unable to jump out of the local optimum after implementing manual intervention strategy 1, the population is considered to have made a mistake in the direction during the evolution of the parent or grandparent. At this time, manual intervention strategy 2 is executed. The backtracking strategy is activated for the current population. All the individuals in the current population, except the optimal individuals, are backtracked to their parents or grandparents. A new evolutionary direction would then be found to strive for a better solution.

**Fig 2:**
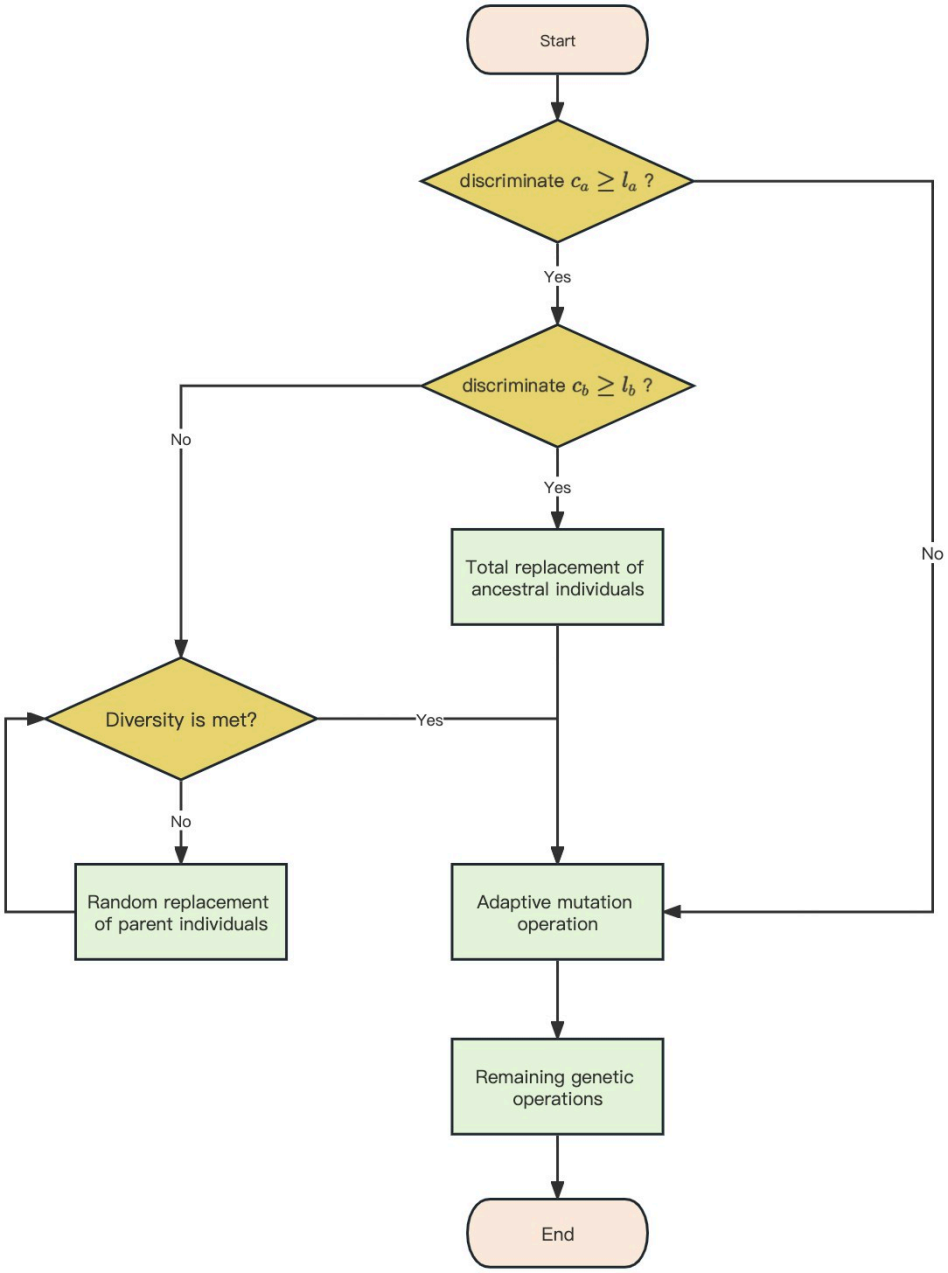
MS-GEP genetic operation.

The flowchart of the MS-GEP genetic operation is shown in Fig 2, and the rest is the same as the standard GEP process.

## 4 Experiment and discussion

In this paper, we conducted two experiments to verify the competitiveness of the proposed MS-GEP in function mining. The experimental environment for the algorithms is Windows 10 operating system with Intel Core i7 3.00 GHz processor and 16 GB RAM. The experimental program is implemented using Python 3.8. The experimental environment for the operational efficiency dataset of map-reduce operations for the catenary monitoring system of high-speed railway is a cluster of three servers. The server parameters are Intel Core i7, 3.00 GHz CPU, and 8 GB RAM. Its operating system is Ubuntu 18.04.2, and its software environment is jdk-1.8.0 and hadoop-2.7.6.

### 4.1 Competitive experiments of the MS-GEP algorithm

To evaluate the proposed MS-GEP algorithm, we created MS-GEP-A, MS-GEP-I, MS-GEP, NMO-SARA [17], GEP [10], and FF-GEP [18] in the literature for comparative experiments. NMO-SARA and MS-GEP-A differ in the mutation rate settings and additional parameters. The additional parameters of NMO-SARA are set the same as those in the literature [17]. MS-GEP-I has the same parameters as GEP, except for la and lb. MS-GEP has the same parameters as FF-GEP, except for mutation rate and additional parameters. The maximum number of evolutionary generations for all algorithms was set to 1000. Table 1 presents the specific parameter settings. All algorithms in the experiment use relative error fitness function. Relative error and the individual fitness *f*_*i*_ are calculated as Eqs (9) and (10), *n* is the number of test samples.

$$\begin{array}{c} RE=\sum_{i=1}^{n}\left|\frac{x_{i}-y_{i}}{y_{i}}\right| \end{array}$$ (9)

$$\begin{array}{c} f_{i}=50\sum_{i=1}^{n}\left(1-\right|\frac{x_{i}-y_{i}}{y_{i}}\left|\right) \end{array}$$ (10)

**Table 1:** Parameters setting of experiment.

	NMO-SARA	MS-GEP-A	GEP	MS-GEP-I	FF-GEP	MS-GEP
Population size	30					
Number of genes	3					
Head length	6					
Linking function	+					
Selection strategy	Roulette-wheel					
Mutation rate	Ref [17]	Eq (5)	0.04	0.04	0.04	Eq (5)
1-point Recombination	0.7	0.7	0.7	0.7	0.7	0.7
Inversion	0.1	0.1	0.1	0.1	0.1	0.1
IS transposition	0.1	0.1	0.1	0.1	0.1	0.1
RIS transposition	0.1	0.1	0.1	0.1	0.1	0.1
Gene transposition	0.1	0.1	0.1	0.1	0.1	0.1
Max Mutation rate	0.1	0.1				0.1
Min Mutation rate	0.01	0.01				0.01
*l*_*a*_				50		50
*l*_*b*_				50		50
Correction factor *α*		0.5				0.5

We employed the test functions in Koza [19], Nguyen [20], and Keijzer [21]. The test samples are generated from 20 groups of test functions. The test functions are shown in Table 2. When the test functions are F1, F2, F3, F4, F5, and F6, the set of algorithmic functions is {+, −, *, /, sin, cos, *exp*, ln}. Meanwhile, when the test functions are F7, F8, F9, and F10, the set of functions is $\left\{+,*,1/n,-n,\sqrt{n}\right\}$. The terminal set is set to the sign of the independent terminal of the test function. For example, the terminal set is {*x*, *y*} for the test function F6.

**Table 2:** GP problems.

Function	Function Formula	Range
F1	*x*^6^ + *x*^5^ + *x*^4^ + *x*^3^ + *x*^2^ + *x*	[-1,1]
F2	*x*^5^ − 2*x*^3^ + *x*	[-1,1]
F3	ln(*x* + 1) + ln(*x*^2^ + 1)	[0, 2]
F4	*sin*(*x*) + *sin*(*x* + *x*^2^)	[-1,1]
F5	*sin*(*x*) + *sin*(*y*^2^)	[0, 1]
F6	2*sin*(*x*)*cos*(*y*)	[0, 1]
F7	*xy* + *sin*(*x* − 1)(*y* − 1)	[-3,3]
F8	$\text{ln}(x+\sqrt{x^{2}+1})$	[0, 100]
F9	$\frac{x^{3}}{5}+\frac{y^{3}}{2}-y-x$	[-3,3]
F10	*e*^−*x*^*x*^3^*cos*(*x*)*sin*(*x*)(*cos*(*x*)*sin*^2^(*x*) − 1)	[0, 10]

Performance metrics for the above six algorithms were determined by executing each algorithm 20 times independently for each test function. Table 3 presents the results. The value “avg” represents the algorithm’s average fitness value after running the specified test function 20 times independently. The number of times the best individual’s RE is less than 0.1^6^ after the algorithm has run the test function is the “target.” “Better” refers to how often the average fitness value of the proposed algorithm performs better than the comparison algorithm. “Equal” is the number of times both algorithms perform equally. “Worse” is the number of times the proposed algorithm performs poorly, as measured by its average fitness value. It can be seen that the proposed MS-GEP outperforms FF-GEP in test functions F2, F4, F5, and F6 with the number of hits. Meanwhile, MS-GEP-I outperforms GEP, except in test function F5. Table 4 presents the results of Wilcoxon’s test. It considers root mean square error (RMSE) of all samples. Analyzing the comparative data, MS-GEP-A, MS-GEP-I, and MS-GEP in most test functions outperform NMO-SARA, GEP, and FF-GEP, respectively. MS-GEP-A, MS-GEP-I, and MS-GEP are more stable.

**Table 3:** Performance metrics of comparative experiment.

	NMO-SARA		MS-GEP-A		GEP		MS-GEP-I		FF-GEP		MS-GEP	
	tar	avg	tar	avg	tar	avg	tar	avg	tar	avg	tar	avg
F1	0	928	0	935	0	948	0	951	0	931	0	950
F2	1	827	0	859	0	829	1	855	1	848	2	870
F3	0	968	0	972	0	977	0	972	0	973	0	973
F4	4	988	2	989	2	991	7	991	5	991	12	997
F5	13	997	15	996	11	983	11	997	13	996	16	998
F6	0	978	1	984	0	971	1	972	1	977	3	983
F7	0	629	0	620	0	618	0	655	0	635	0	651
F8	0	974	0	978	0	976	0	977	0	985	0	985
F9	0	463	0	465	0	464	0	459	0	479	0	467
F10	0	327	0	353	0	331	0	353	0	323	0	369
Better			2	8			3	7			4	7
Equal			0	0			1	1			0	2
Worse			2	2			0	2			0	1

**Table 4:** Wilcoxon’s test.

	NMO-SARA vs MS-GEP-A	GEP vs MS-GEP-I	FF-GEP vs MS-GEP
p-value	8.29E-4	0.00218	1.12E-4

If p-value < 0.05, the difference in results between algorithms is significant according to the Wilcoxon’s signed-rank test.

**Fig 3:**
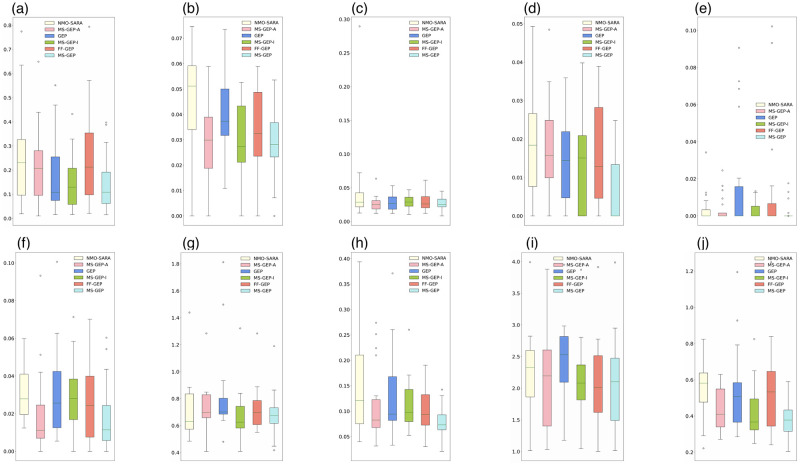
RMSE comparison in all test function (a) F1 (b) F2 (c) F3 (d) F4 (e) F5 (f) F6 (g) F7 (h) F8 (i) F9 (j) F10.

**Fig 4:**
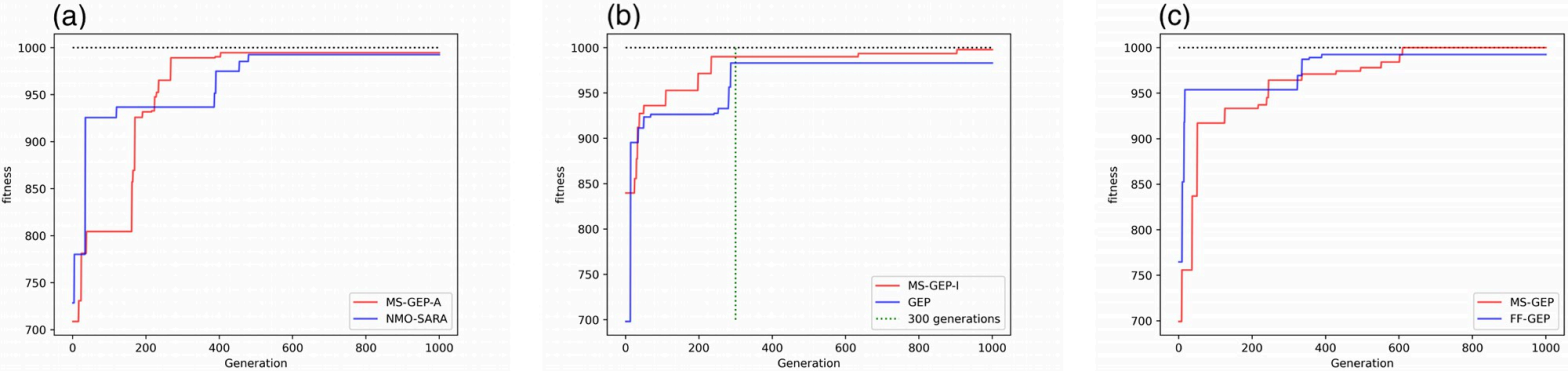
Evolutionary processes on special functions (a) NMO-SARA vs MS-GEP-A (b) GEP vs MS-GEP-I (c) FF-GEP vs MS-GEP.

MS-GEP-A, MS-GEP-I, MS-GEP, GEP, NMO-SARA, and FF-GEP were further analyzed in terms of the maximum and minimum RMSE. As shown in Fig 3, the vertical coordinates are the RMSE. Smaller RMSE means better algorithm performance. Under most of the test functions, MS-GEP-A, MS-GEP-I, and MS-GEP have better maximum and minimum RMSE than NMO-SARA, GEP, and FF-GEP. This indicates that MS-GEP-A, MS-GEP-I, and MS-GEP have a higher possibility to jump out of the local optimum and converge to a better solution during the evolution process. To visually analyze the performance of each algorithm in the dilemma of the local optimum, the change of in the population fitness in the evolutionary process is recorded on the test function F6. As shown in Fig 4(a), NMO-SARA performs significantly better than MS-GEP in the early stage. MS-GEP-A tends to enter the local optimum. MS-GEP-A finally converges to almost the same level as NMO-SARA through the evolution of adaptive mutation rate based on population information entropy. As shown in Fig 4(b), MS-GEP-I and GEP converge to almost the same level when the population evolves to 300 generations. During the evolution of the population after 300 generations, GEP is unable to obtain a better solution, whereas MS-GEP-I finally converges to a better solution by jumping out of the local optimum twice through the manual intervention strategy. As shown in Fig 4(c), FF-GEP enters the local optimum twice for a long time. In contrast, MS-GEP is smoother in convergence, without falling into the local optimum for a long time, eventually converging to the global optimal solution in the middle and late stages of the evolution.

However, MS-GEP-A, MS-GEP-I, and MS-GEP do not outperform GEP, NMO-SARA, and FF-GEP respectively in all test functions. As shown in Fig 3(j), the minimum RMSE of MS-GEP-A in test function F10 is worse than that of NMO-SARA. The GEP algorithm itself has some randomness in the evolutionary process, whereas MS-GEP-A and NMO-SARA are adaptive algorithms whose evolutionary process is not reversible, causing the phenomenon that the minimum RMSE of MS-GEP-A is worse. In addition, as can be seen from Fig 3(d), MS-GEP-I is significantly worse than GEP in terms of the maximum RMSE of the test function F4. The GEP algorithm has some randomness in initializing the population. Although MS-GEP-I and MS-GEP use a manual intervention strategy, the algorithms can change the evolutionary direction and intervene in the population individuals during the evolution process, and the low quality of the initialized population individuals still affects the RMSE of the optimal individuals converged by the algorithms. Similarly, it can also explains that why MS-GEP-I in Fig 3(h) is worse than GEP in the filed of the minimum RMSE and why MS-GEP in Fig 3(i) is worse than FF-GEP in terms of the minimum RMSE and the maximum RMSE.

**Fig 5:**
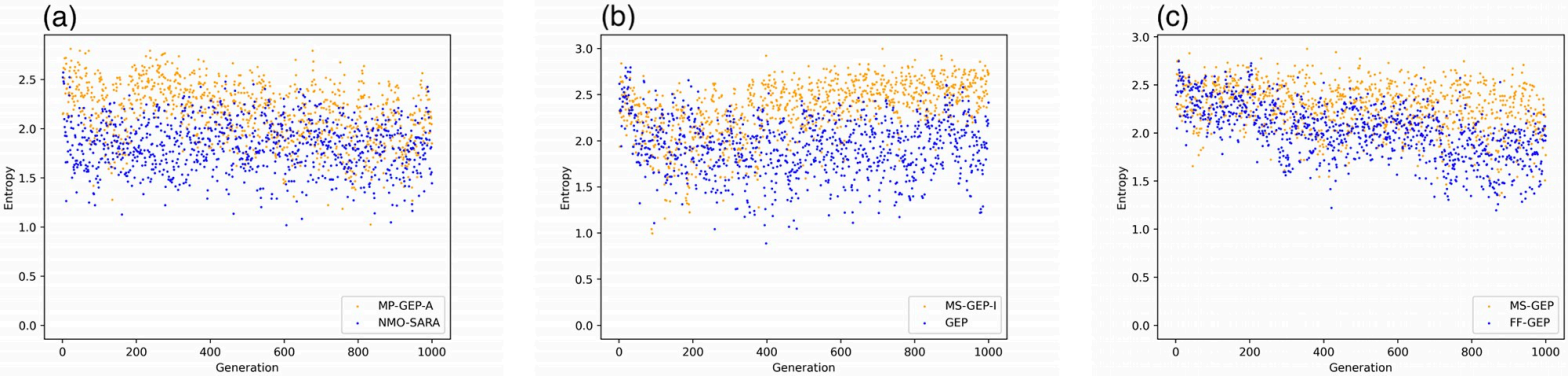
Population diversity comparison on all function (a) NMO-SARA vs MS-GEP-A (b) GEP vs MS-GEP-I (c) FF-GEP vs MS-GEP.

In most test functions, MS-GEP-A, MS-GEP-I, and MS-GEP respectively outperform NMO-SARA, GEP, and FF-GEP. To analyze the change of population diversity of MS-GEP-A, MS-GEP-I, and MS-GEP on the better-performing test functions, we calculate the population information entropy during the evolution of each algorithm according to Eq (2). As shown in Fig 5, the orange dots represent the proposed algorithms in this paper, and the blue dots the comparison algorithms. Fig 5(a) shows the results of running on the test function F10, and Fig 5(b) and 5(c) show the results of running on the test function F7. As shown in Fig 5(a), MS-GEP-A outperforms NMO-SARA in terms of the population information entropy throughout the evolutionary process. It proves that taking evolutionary generations, population diversity, and the quality of individuals in the population into consideration in the adaptive mutation rate setting is helpful to improve the diversity of the population, so as to find a better solution. As shown in Fig 5(b), MS-GEP-I has close population diversity to GEP in the early stage of population evolution. In the middle of the evolution, MS-GEP-I has greater population diversity than GEP. This is also similar to the case of Fig 5(c). This is because the algorithm randomly replaces individuals in the population during the evolution guided by the manual intervention, improving the population diversity and increasing the possibility of the algorithm to find a better solution.

### 4.2 Function mining of map-reduce job CP-EE for high-speed railway systems

#### 4.2.1 Experimental data and modeling

Due to the different operating environments and requirements for system performance, as presented in Table 5, 12 parameters are screened from the literature [22, 23] that have a large impact on the map-reduce job execution efficiency of the high-speed railway system. We set different values for the parameters in Table 5 and ran the Map-Reduce job through an automated test script. The single set of data output is a mapping of 12 configuration parameters and job execution time, where the 12 parameters are the independent variables and the job execution time is used as the dependent variable. A total of 1800 datasets, obtained from the high-speed railway system cluster test, are used to build the map-reduce job CP-EE model, with 1200 and 600 training and test sets, respectively.

**Table 5:** Relationship of map-reduce job configuration parameters.

Parameters	Value	Range
InputDataSize	*x*_0_	[1, 5]
mapreduce.task.io.sort.mb	*x*_1_	[80, 200]
mapreduce.map.sort.spill.percent	*x*_2_	[0.5,0.9]
mapreduce.task.io.sort.factor	*x*_3_	[10, 100]
mapreduce.reduce.shuffle.parallelcopies	*x*_4_	[5, 10]
mapreduce.reduce.shuffle.input.buffer.percent	*x*_5_	[0.5,0.8]
mapreduce.reduce.shuffle.merge.percemt	*x*_6_	[0.5.0.9]
mapreduce.map.output.compress	*x*_7_	0 or 1
mapreduce.output.fileoutputformat.compress	*x*_8_	0 or 1
mapreduce.job.reduce.slowstart.completedamps	*x*_9_	[0.05,0.2]
mapreduce.reduce.merge.immem.threshold	*x*_10_	[10, 1000]
dfs.blocksize	*x*_11_	[128, 1024]

We go on to explain the importance of the parameters in Table 5. *x*_2_, *x*_5_ and *x*_6_ represent the proportion of the corresponding resources allocated to the operation in question. If we set too large, the processing speed of this stage is increased, but more resources are consumed to affect the processing speed of the remaining operations. *x*_3_, *x*_4_ and *x*_10_ represent the handling capacity of the operation and have the same impact as the previous three parameters. *x*_1_ is the size of the buffer, which is based on memory and therefore affects the memory usage of the rest of the tasks. *x*_7_ and *x*_8_ are settings for file compression at different stages, and compression or not will also affect the execution time of the operation. *x*_9_ is the resource request for the Reduce task after the Map task has reached a specified percentage. *x*_11_ is the size of each data block in HDFS. From the above analysis it can be seen that the search for a balance between the various configuration parameters and the performance of the system is important.

We derived a more accurate map-reduce job CP-EE model by setting the MS-GEP parameters. Table 5 presents the mapping relationship between variables and map-reduce job configuration parameters in MS-GEP. MS-GEP parameters are set, as presented in Table 6. The function set is $F=\{+,-,*,/,pow,\sqrt{,}sin,cos,tan,\text{log}\}$. The terminal set is *T* = {*x*_0_, ⋯, *x*_11_}. The evolutionary generations are set to 20000, the selection strategy uses the roulette wheel algorithm. We used the mean square error (MSE) fitness function. MSE are calculated as Eq (11).

$$\begin{array}{c} MSE=\frac{1}{n}\sum_{n=1}^{n}{\left(x_{i}-y_{i}\right)}^{2} \end{array}$$ (11)

**Table 6:** Parameters setting of function mining experiment.

Parameters	Value
Population size	30
Number of genes	8
Head length	10
Linking function	+
Mutation rate	Eq (5)
1-point Recombination	0.3
2-point Recombination	0.3
Gene Recombination	0.1
IS transposition	0.1
RIS transposition	0.1
Gene transposition	0.1
Stagnation generation threshold *l*_*a*_	100
Stagnation generation threshold *l*_*b*_	100

The optimal individual can be derived after running the algorithm according to the above settings. Table 7 presents the map-reduce job CP-EE model K-expressions.

**Table 7:** The K-expression of CP-EE model.

K-expressions
$\sqrt{.}x_{1}.x_{6}.-.sin.log.+.sin.+./.x_{11}.x_{8}.x_{9}.x_{4}.x_{2}.x_{7}.x_{9}.x_{0}.x_{3}.x_{0}.x_{11}$
$\sqrt{.}+.-.-.+.+.x_{1}.*.-.tan.x_{4}.x_{4}.x_{0}.x_{7}.x_{1}.x_{4}.x_{1}.x_{8}.x_{3}.x_{11}.x_{1}$
+.*x*_0_././.−.*x*_10_.−.*. +.+.*x*_3_.*x*_11_.*x*_9_.*x*_4_.*x*_4_.*x*_9_.*x*_10_.*x*_7_.*x*_7_.*x*_7_.*x*_5_
+.*x*_0_. −.*x*_9_.*Sin*.*.+.*log*./.+.*x*_1_.*x*_11_.*x*_4_.*x*_1_.*x*_2_.*x*_1_.*x*_3_.*x*_7_.*x*_5_.*x*_5_.*x*_10_
+./.*x*_0_./. −.*x*_10_.+.*.+. −.*x*_0_.*x*_9_.*x*_11_.*x*_4_.*x*_4_.*x*_10_.*x*_4_.*x*_3_.*x*_1_.*x*_11_.*x*_6_
+.*x*_7_.+.*. −.*x*_5_.*tan*.*cos*.*tan*.*.*x*_10_.*x*_10_.*x*_0_.*x*_0_.*x*_4_.*x*_10_.*x*_6_.*x*_5_.*x*_7_.*x*_9_.*x*_5_
/.+.*x*_2_.*x*_8_. −.+.*x*_8_.*x*_6_.+.*.*x*_0_.*x*_9_.*x*_4_.*x*_11_.*x*_1_.*x*_11_.*x*_9_.*x*_10_.*x*_11_.*x*_9_.*x*_5_
*.+. −.*pow*.*x*_0_.+.*.*cos*.*.*cos*.*x*_2_.*x*_9_.*x*_8_.*x*_2_.*x*_2_.*x*_4_.*x*_0_.*x*_3_.*x*_2_.*x*_4_.*x*_0_

One row represents one gene, eight rows have a total of eight genes. Genes are converted to expression trees by Section 2 and then to algebraic expressions.

#### 4.2.2 Model testing and comparative analysis with other models

Bei et al. [9] constructed Hadoop performance prediction models in stages using a RF algorithm. Were K et al. [24] introduced common methods for constructing complex functional relationships, such as neural networks and support vector machines.

We used three metrics, namely, mean square error (MSE), mean absolute error (MAE), and coefficient of determination (*R*^2^) to test the accuracy and feasibility of the model, according to the calculation by Eqs (11)–(13), respectively. It can be seen that when the predicted value of the model is closer to the target value of the test data, MSE and MAE are smaller, *R*^2^ is larger, and the accuracy of the model is higher.

$$\begin{array}{c} MAE=\frac{1}{n}\sum_{n=1}^{n}\left|x_{i}-y_{i}\right| \end{array}$$ (12)

$$\begin{array}{c} R^{2}=\frac{{\left[\sum_{n=1}^{n}{\left(x_{i}-\bar{x}\right)}^{2}{\left(y_{i}-\bar{y}\right)}^{2}\right]}^{2}}{\sum_{n=1}^{n}{\left(x_{i}-\bar{x}\right)}^{2}{\left(y_{i}-\bar{y}\right)}^{2}} \end{array}$$ (13)

We used support vector regression (SVR) [25], RF [26], back propagation neural network (BPNN) [27], and MS-GEP to model the map-reduce job CP-EE, respectively. SVR, RF and BPNN were trained and tested on the same datasets as MS-GEP. The relevant datasets are set up as in section 4.2.1. MSE, MAE, and *R*^2^ were then calculated based on the test dataset presented in Table 8.

**Table 8:** Performance metrics of function mining experiment.

	MSE	MAE	*R* ^2^
SVR	17.53	3.20	0.787
BPNN	17.28	3.03	0.790
RF	10.85	2.26	0.862
MS-GEP	9.29	2.15	0.883

**Fig 6:**
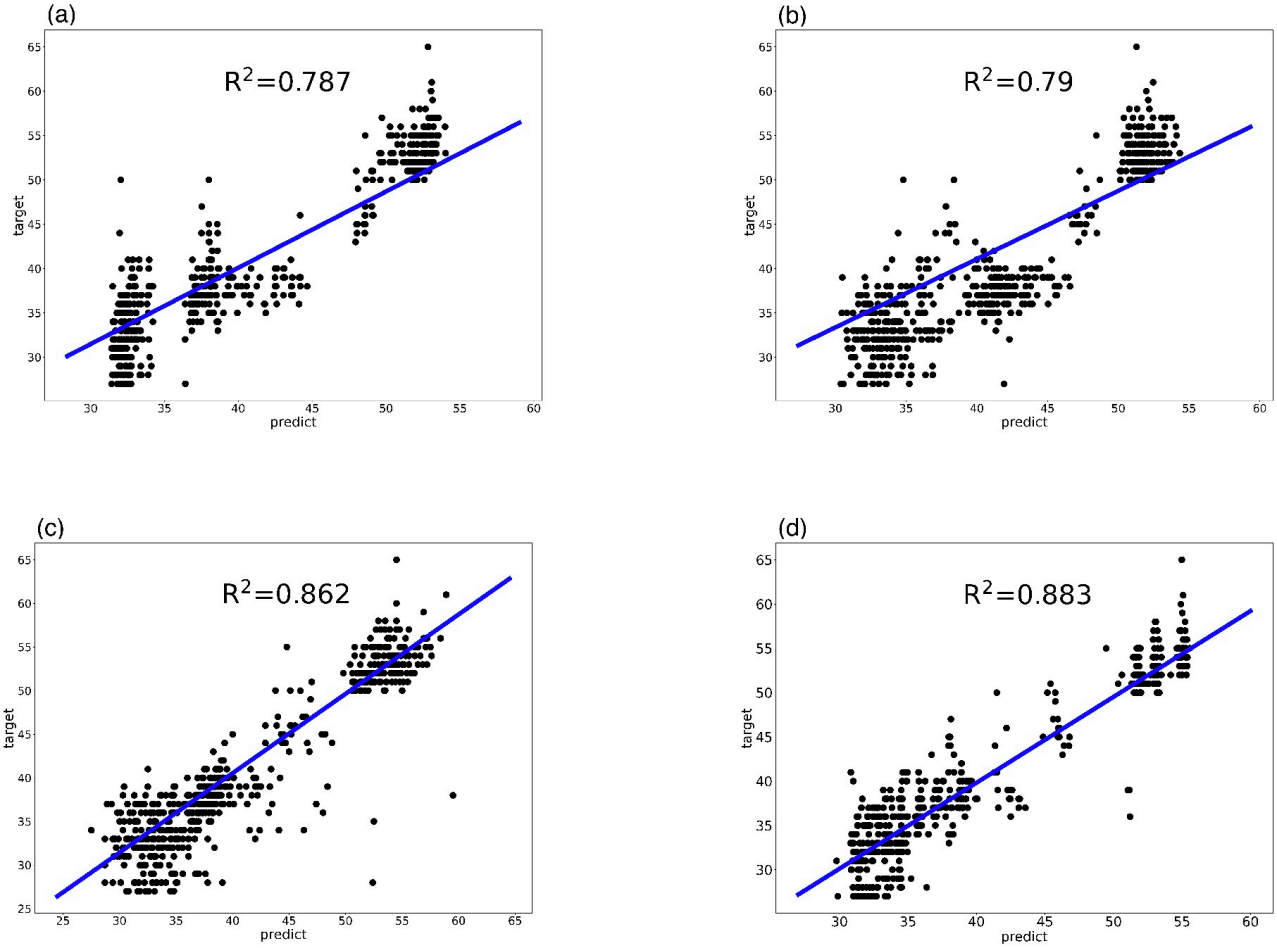
Performance comparison in CP-EE model (a) SVR model (b) BPNN model (c) RF model (d) MS-GEP model.

Fig 6 shows the prediction results of the models developed using SVR, RF, BPNN, and MS-GEP through the test data compared with the target results.

We compared Fig 6(a)–6(d). It can be seen that the prediction results of RF and MS-GEP are closer to the target results. While the predictions of SVR and BPNN are relatively discrete, so RF and MS-GEP fit better models with higher model accuracy than the models fitted with SVR and BPNN. Specifically, the predicted values of SVR and BPNN are more dispersed from the regression line between 50 and 55, which may be due to the fact that SVR and BPNN do not take certain variables into account better in their modeling. As can be seen in the results for RF and msgpe where *R*^2^ is closer, RF is more discrete than MS-GEP for predictions with target values of 45 or less. This difference may account for the slightly lower *R*^2^ of RF than MS-GEP.

As presented in Table 8, RF and MS-GEP are closer in the three indicators of MSE, MAE, and *R*^2^, indicating that the model established by MS-GEP is reliable. The three indexes of MS-GEP are better than RF, so the model of map-reduce job CP-EE established by MS-GEP is better than that of the RF. Table 9 shows that MSGEP outperforms RF in all but the minimum error. The MAE shows that MS-GEP is more accurate, while the standard deviation demonstrates that the MS-GEP performance is more stable. However, the minimum error of RF is better than that of MS-GEP, which may be due to the complexity of the mathematical expression fitted by MS-GEP, so it is difficult to come up with an exact error of zero.

**Table 9:** The test error of RF and MS-GEP.

	RF	MS-GEP
MAE	2.26	2.15
maximum error	20.40	15.21
minimum error	0	0.006
standard deviation	2.28	2.08

## 5 Conclusion

This paper proposed a new algorithm, i.e., MS-GEP, based on the shortcomings of the standard GEP algorithm. Based on the theoretical analysis, we set the adaptive mutation rate based on the population information entropy, individual fitness, and evolutionary generations. Inspired by manual intervention in biological evolution, we create a manual intervention strategy to guide the population evolution. The results show that the proposed MS-GEP improves population diversity, avoids premature convergence, and fall into local optimum.

Benchmark tests have shown that the proposed MS-GEP has better solution quality and higher stability than NMO-SARA, GEP and FF-GEP in most cases. In the experiments of high-speed railway map-reduce job configuration parameters function mining, the proposed MS-GEP still outperforms other commonly used algorithms in constructing complex functions in each evaluation index when a specific function model is available, indicating its effectiveness in solving real-world problems.

However, we also recognized that map-reduce job configuration parameters are characterized by nonlinearity and high noise, which may have some impact on model performance. In future research, we will aim to reduce these disturbances more comprehensively and effectively to further improve the accuracy and robustness of the model. In addition, we will also explore the application of MS-GEP to other fields to expand its application scope.
